# Male with an apparently normal phenotype carrying a *BRCA1* exon 20 duplication *in trans* to a *BRCA1* frameshift variant

**DOI:** 10.1186/s13058-023-01755-9

**Published:** 2024-01-09

**Authors:** Ines Block, Àngels Mateu-Regué, Thi Tuyet Nhu Do, Ieva Miceikaite, Daniel Sdogati, Martin J. Larsen, Qin Hao, Henriette Roed Nielsen, Susanne E. Boonen, Anne-Bine Skytte, Uffe Birk Jensen, Louise K. Høffding, Arcangela De Nicolo, Alessandra Viel, Emma Tudini, Michael T. Parsons, Thomas V. O. Hansen, Maria Rossing, Torben A. Kruse, Amanda B. Spurdle, Mads Thomassen

**Affiliations:** 1 Department of Clinical Genetics, Odense University Hospital, Odense, Denmark; 2 Institute of Pharmacology and Clinical Pharmacy, University of Marburg, Marburg, Germany; 3 Center for Genomic Medicine, Rigshospitalet, Copenhagen University Hospital, Copenhagen, Denmark; 4 Clinical Genome Center, Human Genetics, Department of Clinical Research, University of Southern Denmark, Odense, Denmark; 5 Lundbeckfonden Center of Excellence NanoCAN, Institute of Molecular Medicine, University of Southern Denmark, Odense, Denmark; 6 Molecular Oncology, Institute of Molecular Medicine, University of Southern Denmark, Odense, Denmark; 7 Department of Clinical Genetics, Aarhus University Hospital, Aarhus, Denmark; 8 Center for Clinical Genetics and Genomic Diagnostics, Zealand University Hospital, Roskilde, Denmark; 9 Center for Omics Sciences, IRCCS San Raffaele Scientific Institute, Milan, Italy; 10 Unit of Functional Oncogenetics and Genomics, Centro Di Riferimento Oncologico Di Aviano (CRO) IRCCS, Aviano, (PN) Italy; 11 Population Health Program, QIMR Berghofer Medical Research Institute, Herston, Brisbane, Australia; 12 Department of Clinical Genetics, Rigshospitalet, Copenhagen University Hospital, Copenhagen, Denmark; 13 Department of Clinical Medicine, Faculty of Health and Medical Sciences, University of Copenhagen, Copenhagen, Denmark

**Keywords:** *BRCA1*, Dual carrier, Fanconi Anemia, Variant classification, Exon duplication, Transcription activation domain assay

## Abstract

**Background:**

Reports of dual carriers of pathogenic *BRCA1* variants *in trans* are extremely rare, and so far, most individuals have been associated with a Fanconi Anemia-like phenotype.

**Methods:**

We identified two families with a *BRCA1* in-frame exon 20 duplication (Ex20dup). In one male individual, the variant was *in trans* with the *BRCA1* frameshift variant c.2475delC p.(Asp825Glufs*21). We performed splicing analysis and used a transcription activation domain (TAD) assay to assess the functional impact of Ex20dup. We collected pedigrees and mapped the breakpoints of the duplication by long- and short-read genome sequencing. In addition, we performed a mitomycin C (MMC) assay from the dual carrier using cultured lymphoblastoid cells.

**Results:**

Genome sequencing and RNA analysis revealed the *BRCA1* exon 20 duplication to be in tandem. The duplication was expressed without skipping any one of the two exon 20 copies, resulting in a lack of wild-type transcripts from this allele. TAD assay indicated that the Ex20dup variant has a functional level similar to the well-known moderate penetrant pathogenic *BRCA1* variant c.5096G > A p.(Arg1699Gln). MMC assay of the dual carrier indicated a slightly impaired chromosomal repair ability.

**Conclusions:**

This is the first reported case where two *BRCA1* variants with demonstrated functional impact are identified *in trans* in a male patient with an apparently normal clinical phenotype and no *BRCA1-*associated cancer. The results pinpoint a minimum necessary BRCA1 protein activity to avoid a Fanconi Anemia-like phenotype in compound heterozygous status and yet still predispose carriers to hormone-related cancers. These findings urge caution when counseling families regarding potential Fanconi Anemia risk. Furthermore, prudence should be taken when classifying individual variants as benign based on co-occurrence *in trans* with well-established pathogenic variants.

**Supplementary Information:**

The online version contains supplementary material available at 10.1186/s13058-023-01755-9.

## Background

Inherited *BRCA1* and *BRCA2* variants are the main known cause of hereditary breast and ovarian cancer cases. While mono-allelic pathogenic *BRCA1* variants are relatively common in the general population worldwide, biallelic pathogenic *BRCA1* variants are rarely reported and, until recently, were assumed to be lethal during embryogenesis [1]. However, it has been suggested that dual carriers can survive due to several mechanisms, including some degree of retained wild-type activity from at least one allele or rescue mechanisms [1–3]. Nevertheless, these patients are still likely to develop early onset cancer and are often characterized by congenital anomalies and potentially chromosomal fragility. Previous reports of dual *BRCA1* carriers are reviewed in Table 1.

**Table 1:** Comparison of biallelic *BRCA1* variant carriers regarding their genotype, phenotype, rescue mechanisms and classification of the detected variants^1^

Study	Variant 1 (HGVS) Variant 2 (HGVS)	Physical phenotype (onset age in years)	Chromosome breakage	Comment including potential protein function from variant allele, e.g., missense or rescuing in-frame isoform	Historical ENIGMA classification^2^, or ClinVar classifications	Rationale for classification
Seo et al. [1]	c.1115G > A, p.(Trp372*) c.1115G > A, p.(Trp372*)	Sibs ♀, FA, T-ALL (5y), ♀, FA, NC (8y)	Sib 1—DEB sensitive and spontaneous; Sib 2—elevated chromosomal sensitivity to DEB and MMC	∆11q ∆11q	C5 C5	Variant alleles predicted to encode a truncated non-functional protein
Seo et al. [1]	c.1292 T > G, p.(Leu431*) c.1292 T > G, (p.Leu431*)	Sibs: ♀, FA, NB (2y), ♂, FA, NC (15.5y)	Both sibs, DEB sensitive and spontaneous	∆11q ∆11q	C5 C5	Variant alleles predicted to encode a truncated non-functional protein
Sawyer et al*.* [4]	c.594_597del, p.(Ser198Argfs*35) c.5095C > T, p.(Arg1699Trp)	♀, FA, BC (23y)	DEB and MMC sensitive	∆9–10, and ▼10p (r.594-21_594-1ins) missense	n.a C5	c.594_597del results in a truncation in BRCA1 exon 10 (de la Hoya et al*.* [29]), where ∆9–10 is shown to be naturally occurring isoform c.5095C > T: IARC C5 based on posterior probability from multifactorial likelihood analysis, thresholds for class as per Plon et al*.* [30]. Posterior probability = 1 (Lindor et al*.* [31])
Domchek et al*.* [2]	c.2457delC, p.(Asp825Glufs*21) c.5207 T > C, p.(Val1736Ala)	♀, FA, OC (28y)	Not tested	∆11q missense	C5 C5	c.2457delC allele predicted to encode a truncated non-functional protein c.5207T > C: IARC C5 based on posterior probability from multifactorial likelihood analysis, thresholds for class as per Plon et al*.* [30]. Posterior probability = 0.9998
Freire et al*.* [7]	c.2709 T > A, p.(Cys903*) c.2709 T > A, p.(Cys903*)	♀, FA, NC (3.7y)	DEB sensitive and spontaneous	∆11q ∆11q	n.a n.a	Variant alleles predicted to encode a truncated non-functional protein
Keupp et al*.* [3]	c.181 T > G, p.(Cys61Gly) c.5096G > A, p.(Arg1699Gln)	♀, mild FA, BC (30y)	DEB‐induced chromosome fragility in patient-derived blood lymphocytes within normal range	p.(Cys61Gly) moderate penetrance missense	C5 C5	c.181T > G IARC C5 based on posterior probability from multifactorial likelihood analysis, thresholds for class as per Plon et al*.* [30]. Posterior probability = 1 (Lindor et al*.* [31]) The c.5096G > A variant has been shown to impact function [22, 32–35]. A genetic study ([16]) reported it to be associated with reduced risk compared to another pathogenic missense substitution variant at the same residue (BRCA1 c.5095C > T p.(Arg1699Trp)). A subsequent larger genetic study including 129 families ([17]) reported HRs of 2.83 for breast cancer and 5.83 for ovarian cancer risk, and estimated the cumulative risk to age 70 to be 20% for breast cancer and 6% for ovarian cancer. This variant is considered pathogenic with reduced penetrance relative to the average BRCA1 truncating pathogenic variant
Chirita-Emandi et al*.* [8]	c.2933dupA, p.(Tyr978*) c.843_846delCTCA, p.(Ser282TyrfsTer15)	♂ FA, NC (2y)	DEB and MMC sensitive	∆11q ∆11q	n.a C5	Variant alleles predicted to encode a truncated non-functional protein
Kwong et al*.* [36]	c.4065_4068delTCAA, p.(Asn1355Lysfs*10) c.5406 + 7A > G, p.?	♀, no or very subtle FA features, OC (43y), BC (44y)	No definitive diagnostic test for FA was performed due to a lack of clinical indication of FA-like features being observed	∆11q Partial effect on splicing (not quantified)	C5 n.a. (likely benign in ClinVar	c.4065_4068delTCAA predicted to encode a truncated non-functional protein (ENIGMA Rules, Version 2.5.1, 29 June 2017) For c.5406 + 7A > G, the authors analyzed cDNA from blood and identified cryptic splicing with deletion of 74 nucleotides from transcript and predicted frameshift. However, the RNA result was not quantitated. No other quantitative studies of the variant are available
Borlin et al*.* [37]	c.1116G > A, p.(Trp372*) c.5017_5019del, p.(His1673del)	♀, FA, CNS (1y)	MMC-induced chromosomal breakage analysis in peripheral blood lymphocytes showed strongly reduced proliferation upon stimulation, but no evidence of increased chromosomal breakage	∆11q In-frame deletion	C5 Clinvar C3, C4	c.1116G > A allele predicted to encode a truncated non-functional protein c.5017_5019del considered pathogenic by 8/13 submitters in Clinvar
This study	c.2475delC, p.(Asp825Glufs*21) Ex20dup	♀, no FA symptoms, LC (64y)	MMC within normal range, Lymphoblastic cell line	∆11q no exon 20 skipping	C5 C3	c.2457delC allele predicted to encode a truncated non-functional protein Ex20dup function similar to p.(Arg1699Gln) indicating possibly moderate penetrance. See discussion for ACMG classification

1: Only cases with at least one confirmed likely pathogenic or pathogenic variant and features of FA OR both alleles considered risk associated and not necessarily symptoms or MMC/DEB results indicating FA are included

2: Classification as per ENIGMA historical rules version 2.5.1, 29 June 2017 unless otherwise specified

*FA* Fanconi anemia-like disorder, *T-ALL* T-cell Acute Lymphoblastic Leukemia, *OC* Ovarian Cancer, *NB* Neuroblastoma, *BC* Breast Cancer, *CLL* Chronic Lymphatic Leukemia, *LC* Lung Cancer, *NC* No cancer at last follow-up, *CNS* Central nervous system tumor *FL* full length transcript, *Δ* skipping of reference exonic sequences *▼* inclusion of reference intronic sequences, *q* donor shift, *DEB* Diepoxybutane assay, *MMC* Mitomycin C

n.a.: variant not classified by ENIGMA

Previously reported variants with residual function from dual *in trans BRCA1* carriers include at least one variant with reduced penetrance or potential rescue mechanisms resulting in some level of BRCA1 function. In some studies, one of the variants was a missense variant that might retain some activity. For example, Domchek et al*.* reported a patient with early onset ovarian cancer who had a frameshift variant on one allele and a *BRCA1* missense variant (c.5207T > C p.(Val1736Ala)) *in trans* [2]. They suggested that p.(Val1736Ala) was likely pathogenic but had sufficient residual BRCA1 activity allowing embryonal development and viability through adulthood. However, the authors assumed that the biallelic *BRCA1* variants caused phenotypical differences and developmental delay in the patient, which they diagnosed as a “Fanconi Anemia (FA)-like phenotype” [2]. Similar results were reported by Sawyer et al. [4] and Keupp et al*.* [3]. In agreement with the idea that missense variants may retain some activity, a recent study showed reduced penetrance for patients above 50 years of age carrying pathogenic missense variants compared to those carrying protein truncating variants [5].

Protein truncating variants located in exon 11 (according to legacy exon numbering) of *BRCA1* have been shown to retain minimal protein function since a naturally occurring alternative splice donor site results in the expression of an in-frame transcript lacking most of the exon [6]. Freire et al*.* [7], Chirita-Emandi et al*.* [8], and Seo et al. [1] also reported a total of six patients with biallelic protein truncating *BRCA1* variants in exon 11. They all presented with features of FA and two of the patients developed cancers at 2 and 5 years age.

Here, we report a male lung cancer patient with apparently no FA features who was tested for *BRCA1* variants due to a family history of breast, ovarian, and prostate cancer. We identified a pathogenic frameshift variant (c.2475delC) in *BRCA1* exon 11, likely responsible for the familial cancer history and a duplication of *BRCA1* exon 20 (Ex20dup) *in trans*.

We also identified the latter variant in a female patient diagnosed with breast cancer at age 46 from an unrelated family. We therefore focused on determining the functional impact of the *BRCA1* Ex20dup variant to guide patient counseling and clinical management.

## Methods

### Patient material

**Fig. 1:**
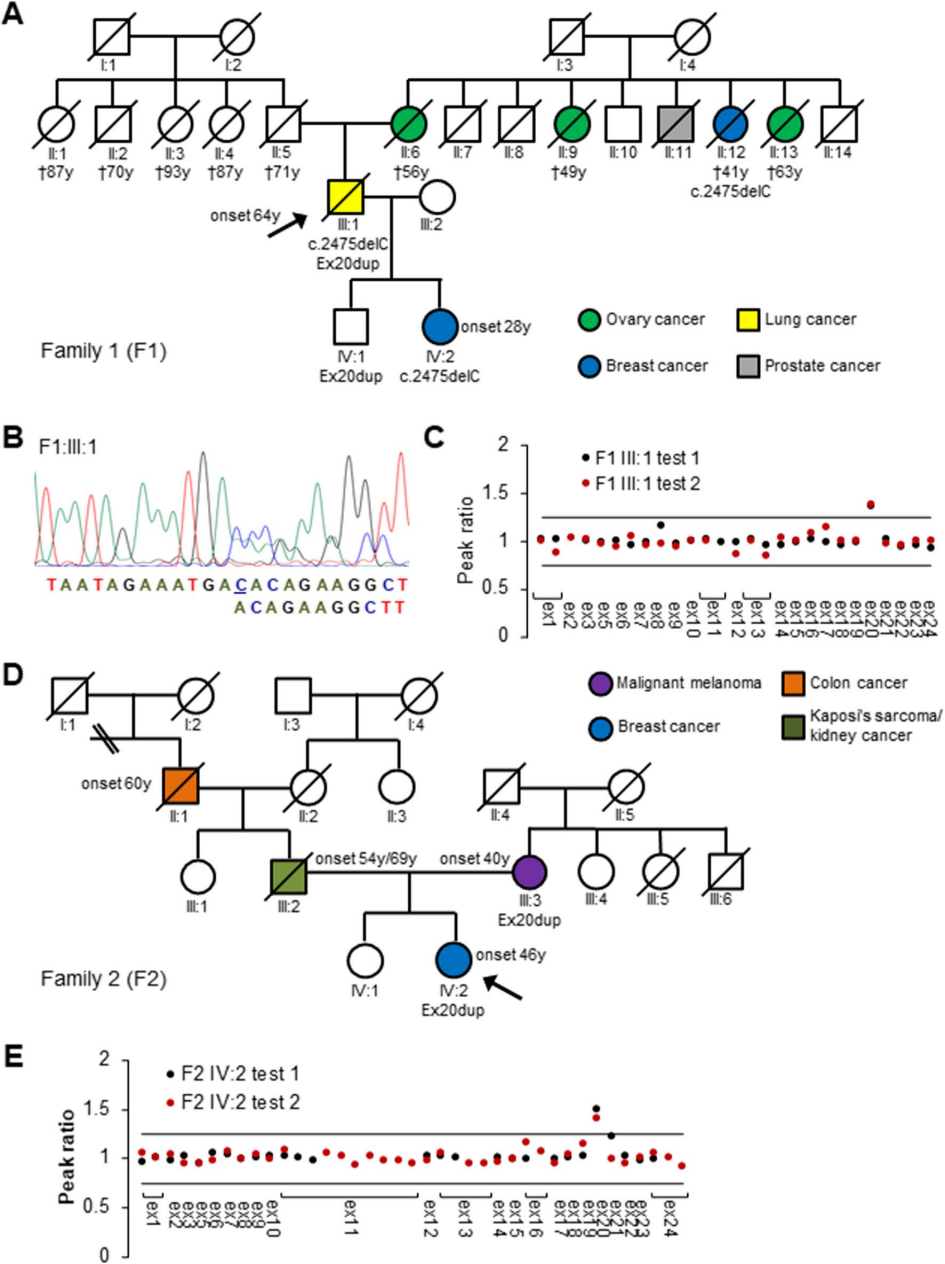
**A** Pedigree of a lung cancer patient F1 III:1 presenting with a *BRCA1* c.2475delC frameshift variant detected by Sanger sequencing (**B)** and an exon 20 duplication (Ex20dup) *in trans* identified by two different MLPA test kits (**C)**. Pedigree of a breast cancer patient F2 IV:2 (**D)** presenting with *BRCA1* Ex20dup as detected by two MLPA kits (**E)**. Probands in pedigrees (F1 III:1 and F2 IV:2) are marked by arrows. Variants detected in family members are indicated below the symbols of the tested individuals. If available, the patient age at disease onset in years is indicated

Blood samples were collected from probands from the two families. A sample from the proband in family 1 (F1) was used for immortalizing lymphocytes (LCL) by Epstein-Barr virus infection. Cells were treated with Puromycin before harvest to prevent nonsense-mediated mRNA decay. In addition, PAXgene® Blood tubes were collected from probands from both families (Fig. 1A, F1 III:1 and Fig. 1D, F2 IV:2).

### Variant detection

Genetic testing was performed using different technologies including Protein Truncation Test, Multiplexed Ligation-dependent Probe Amplification (MLPA) and Sanger sequencing [9].

Sequencing primers were designed to span the breakpoint region of the variant call from Whole Genome Sequencing (WGS) data (chr17: 41,206,829–41,211,992). The following primers were used: 5′-ATGTGATCTGGCCCTCATCT´-3′ intron 19 (61.79 °C), 5′-TAACTGGGCGTGGTGGTAG-3 intron 20 (61.46 °C). These primers were used for both touchdown PCR and Sanger sequencing reaction.

MLPA analysis was performed according to the manufacturer's instructions (MRC Holland, Amsterdam, the Netherlands) using kits P002 and P087 specific for the *BRCA1* gene.

We used exon numbering in *BRCA1* according to the traditional numbering, i.e., with no exon 4. Variants are described in accordance with the HGVS guidelines using the RefSeq transcript identifier NM_007294.4.

### RNA extraction and analysis

Total RNA was extracted from a PAXgene sample using the PAXgene® Blood RNA Kit from PreAnalytiX by Qiagen according to the manufacturer’s protocol. RNA was extracted from LCL of the proband in family 1 (F1 III:1) using the RNeasy Mini Kit (Qiagen) following the instructions of the manufacturer including on-column DNA digest using DNaseI. RNA integrity and yield were subsequently assessed using an Agilent Bioanalyzer and NanoDrop ND-8000 instrument. For cDNA synthesis, the SuperScript III First-Strand Synthesis System from the RT-PCR Kit (Invitrogen, cat. No. 18080-051) was applied. An area spanning from exon 15 to exon 21 was amplified by PCR using the primers: 5′-CAACAGCTGGAAGAGTCTG-3′ and 5′-CCATAGCAACAGATTTCTAGC-3′. PCR products of approximately 700 bp and 800 bp in length were extracted from agarose gel. PCR products were re-amplified using the above noted primers before Sanger sequencing.

### Whole genome sequencing

Long-read WGS was performed using Oxford Nanopore sequencing. High molecular weight DNA was extracted using Nanobind CBB Big DNA Kit (Circulomics). The sequencing library was prepared using a Ligation Sequencing Kit (Oxford Nanopore Technologies) and sequenced on a PromethION (Oxford Nanopore Technology) using a R9.4.1 flow cell. Data were mapped to the GRCh37 (hg19) reference genome using Minimap2. Genome Ribbon was used for visualization of long-read data (https://genomeribbon.com/).

Short-read WGS was performed using Illumina NovaSeq 6000 platform at 2 × 150bp read length. TruSeq™ DNA PCR-Free kit (Illumina, cat.no. 20015963) was used for sequencing library preparation. All processes were done according to the manufacturers’ instructions. Mean sequencing coverage of 30 × was achieved.

WGS data were de-multiplexed and mapped to GRCh37 (hg19) reference genome using BWA for paired-end alignments. SAM files were sorted and converted into BAM files by Picard. BAM files were sorted, indexed and duplicates were marked. Delly2 software was used for variant calling. IGV (Integrative Genomics Viewer) was used for further manual inspection of the region of interest.

### Plasmids

The 5 × GAL4-luciferase reporter plasmid (Sun et al. [10]), pHKG3 (Bannister et al. [11]), and pYFP_BRCA1 (Fabbro et al. [12]) were kind gifts from Richard A. Maurer (Oregon Health Sciences University, Portland, Oregon), Tony Kouzarides (University of Cambridge), and Beric R. Henderson (Westmead Institute for Cancer Research, University of Sydney), respectively, while pRL-0 was obtained from Promega. pBluntII-BRCA11396-1863 was constructed by PCR using pYFP-BRCA1 as a template and the oligonucleotides 5′-TTTTGAATTCTCAACAGAAAGGGTC-3′ and 5′-TACTTATCTAGAGTTAGTAGTGGCTGTGG-3’. pHKG3-GAL4-BRCA1-BRCT1-2 was constructed by fusing a gene fragment coding for amino acids 1396 to 1863 of BRCA1 in-frame to the 3´end of the coding sequence of the DNA binding domain of GAL4 (amino acids 1–147). Finally, pcDNA3.1-GAL4-BRCA1-BRCT1-2 was constructed by cloning of GAL4-BRCA1-BRCT1-2 into pcDNA3.1 (Invitrogen). The pcDNA3.1 GAL4-BRCT1-2 wild-type plasmid was mutated using the QuikChange Lightning Multi Site-Directed Mutagenesis Kit (Agilent) and the following primers: 5′-ATTTCAGTGTCCATTCACACACAAACTCAGCATC-3′ (p.(Arg1699Trp)); 5′-AGTTTGTGTGTGAACAGACACTGAAATATTT-3’ (p.(Arg1699Gln)); 5’-CCTTCACCAACAGGCCCACAGATCAAC-3’ (p.(Met1775Arg)); 5’-CCACCAAGGTCCAAAGTGAGCAAGAGAATC-3’ (p.(Arg1751*)). Successful mutagenesis was verified via Sanger sequencing.

### Cloning of *BRCA1* exon 20 dup variant

cDNA was generated from RNA purified from the F1 proband’s LCL using the RevertAid TM H Minus First-Strand cDNA Synthesis Kit. Using the primers 5’-CACCGAATTCCAGAGGGATACCATGCAACATAAC-3’ and 5’-TCTAGATCAGTAGTGGCTGTGGGGG-3’ a 1491 bp fragment spanning the BRCT1-2 region including the Ex20dup was amplified and cloned into the pENTR™/D-Topo vector using the pENTR Directional TOPO® Cloning kit (Invitrogen). After sequence confirmation via Sanger sequencing (see Additional file 1: Table S1 for sequencing primer sequences), the vector was digested using EcoRI and BsgI (NEB) and the Ex20dup spanning region ligated into the pcDNA3.1 reporter plasmid replacing GAL4-BRCT1-2 wild type.

**Cell lines** HEK293 and T47D cells were purchased from ATCC (American Type Culture Collection) and cultured in high glucose (4.5 g/L) Dulbecco’s Modified Eagle Medium (DMEM GlutaMAX™) supplemented with 1 mM sodium pyruvate (Thermo Fischer), 10% Fetal Bovine Serum (Biowest) and 1% penicillin/streptomycin (Thermo Fischer). Cells were grown at 37°C and 5% CO_2_ in a humidified incubator.

### Transcriptional activation assay

300.000 HEK293 cells or 150.000 T47D cells were seeded in triplicates in 6-well plates. After 24 h, cells were co-transfected using Fugene 6 (6 μl/well) with 2 μg of pcDNA3.1 GAL4-BRCT1-2 wild type or mutated fusion protein variant, 1 μg of a GAL4 Luciferase reporter vector and 0.1 μg of PrLO plasmid for normalization. Cells were incubated for 48 h at 37°C and 5% CO_2_ and Firefly luciferase and Renilla luciferase were measured in a GloMax® 96 Luminometer using the Dual-Luciferase® Reporter Assay Kit (Promega) following the manufacturer’s protocol.

Two-tailed unpaired Student`s t-test was used to compare relative luciferase activities between the different variants. *p* < 0.05 was considered significant.

## Results

The proband, a male patient (Fig. 1A, F1 III:1) was diagnosed with lung cancer (planocelluar carcinoma) at an age of 64 years. He had smoked 20 cigarettes/day since he was 14 years old. Genetic testing revealed two *BRCA1* variants: a frameshift variant c.2475delC (p.(Asp825Glufs*21), Fig. 1B) in exon 11 and a duplication of exon 20 (Fig. 1C). The patient had an apparently normal phenotype with respect to clinical features of FA. As the patient died from the lung cancer, we were unable to do a clinical assessment for Fanconi Anemia, however, going through the patient journal we did not identify even subtle features of FA. MMC assay for chromosomal breakage was performed on an immortalized lymphoblast culture. Zero out of 20 metaphases examined had more than 10 chromosomal breaks in agreement with a normal phenotype. Nevertheless, an average of 2.9 breaks was observed compared to 0.6 in a control LCL cell line (data not shown). The patient underwent surgery for lung cancer but did not receive adjuvant therapy, since no lymph node metastases were detected. However, one year later a metastasis was detected in the lung. He received three rounds of carboplatin and vinorelbine. During the treatment, he experienced low grade neuropathy in the hands and fatigue but otherwise few adverse effects.

The maternal branch of the family was strongly affected by cancer; the patient’s mother (II:6) died of bilateral ovarian cancer by the age of 56, three maternal aunts died of breast cancer (II:12) or ovarian cancer (II:9; II:13) at the age of 41, 49 and 63, respectively, and an uncle had prostate cancer (II:11). Clinical information from the paternal family was sparse; however, the father and his four siblings all lived until 70–93 years of age (Fig. 1A, II:1–II:5). The death certificate from the father indicated no cancer. The proband’s daughter (IV:2) tested positive for the c.2475delC variant and was tested negative for the Ex20dup variant (MLPA data not shown). She was diagnosed with breast cancer at the age of 28. Her grandaunt (II:12) also carried c.2475delC but not Ex20dup as confirmed by MLPA (data not shown). The proband’s son (IV:1) inherited the Ex20dup variant (Fig. 1A) and is to date disease-free at the age of 49. As the variants were separately passed on to the daughter and the son of the patient (Fig. 1A), the variants were confirmed to be *in trans*.

In another family (F2, Fig. 1D), *BRCA1* Ex20dup was identified in a female who developed breast cancer (invasive ductal carcinoma, estrogen receptor positive (100%), HER2 normal expression) at 46 years (Fig. 1E, F2 IV:2). The family history of this patient did not provide obvious evidence for hereditary breast and ovarian cancer syndrome (HBOC).

A third, Italian, family was previously reported [13] in which the *BRCA1* Ex20dup was identified in a patient diagnosed with early onset breast cancer (HER2 negative; hormone receptor status unknown), who succumbed to the disease at age 34. The updated family history included a total of four additional breast cancer cases: the proband’s mother diagnosed at age 55, one maternal aunt diagnosed at 50, and another maternal aunt and her daughter (a first cousin of the proband’s), diagnosed at 74 and 54, respectively. Cascade testing could not be extended to any of the family members with breast cancer, but it was offered to two unaffected first-degree blood relatives, the proband’s daughter and sister, yielding negative results. Family history also included a lung cancer diagnosis in the proband’s maternal grandfather. No further clinical or pathological information could be retrieved.

**Fig. 2:**
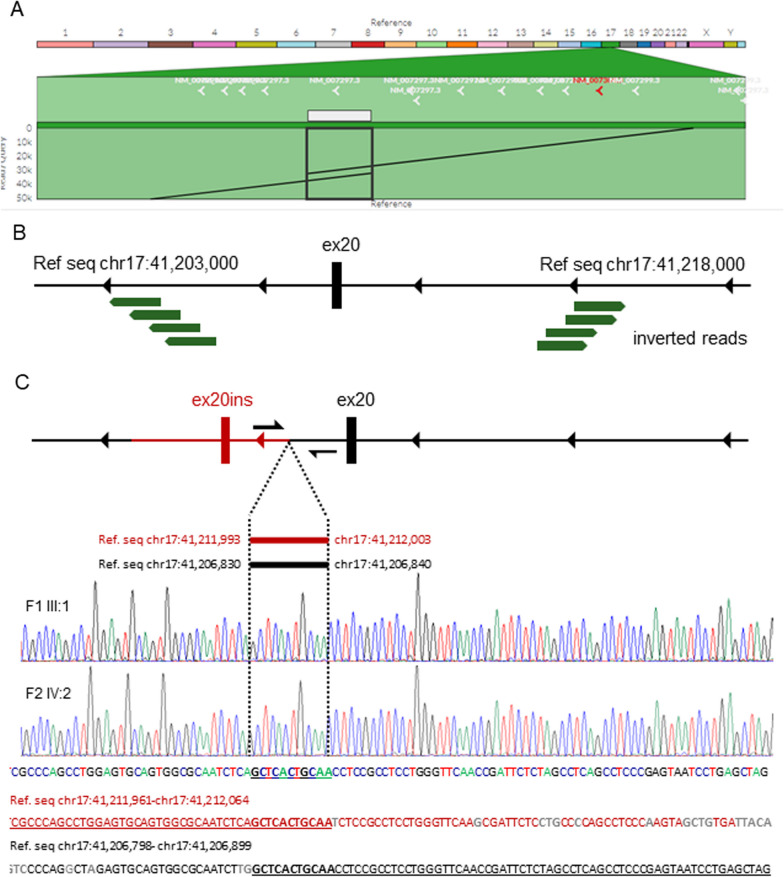
Schematic illustration of the breakpoint region identified via Oxford Nanopore and Illumina whole-genome sequencing and Sanger sequencing. **A** Oxford Nanopore long-read sequencing confirmed in-frame exon 20 duplication. **B** Multiple inverted Illumina reads fine-mapped the duplication to chr17:41,203,000-chr17:41,218,000. **C** Sanger sequencing of the PCR-amplified amplicon junction (primer locations are indicated by the black half arrows) aligned to the *BRCA1* reference sequence. Eleven consistent bp between chr17:41,206,830-chr17:41,206,840 and chr17:41,211,993-chr17:41,212,003 were identified flanked by corresponding sequences of intron 19 and intron 20. The ref. seq of intron 19 is shown in red and the ref. seq of intron 20 is shown in black. Non-matching bases are displayed in light gray

**Fig. 3:**
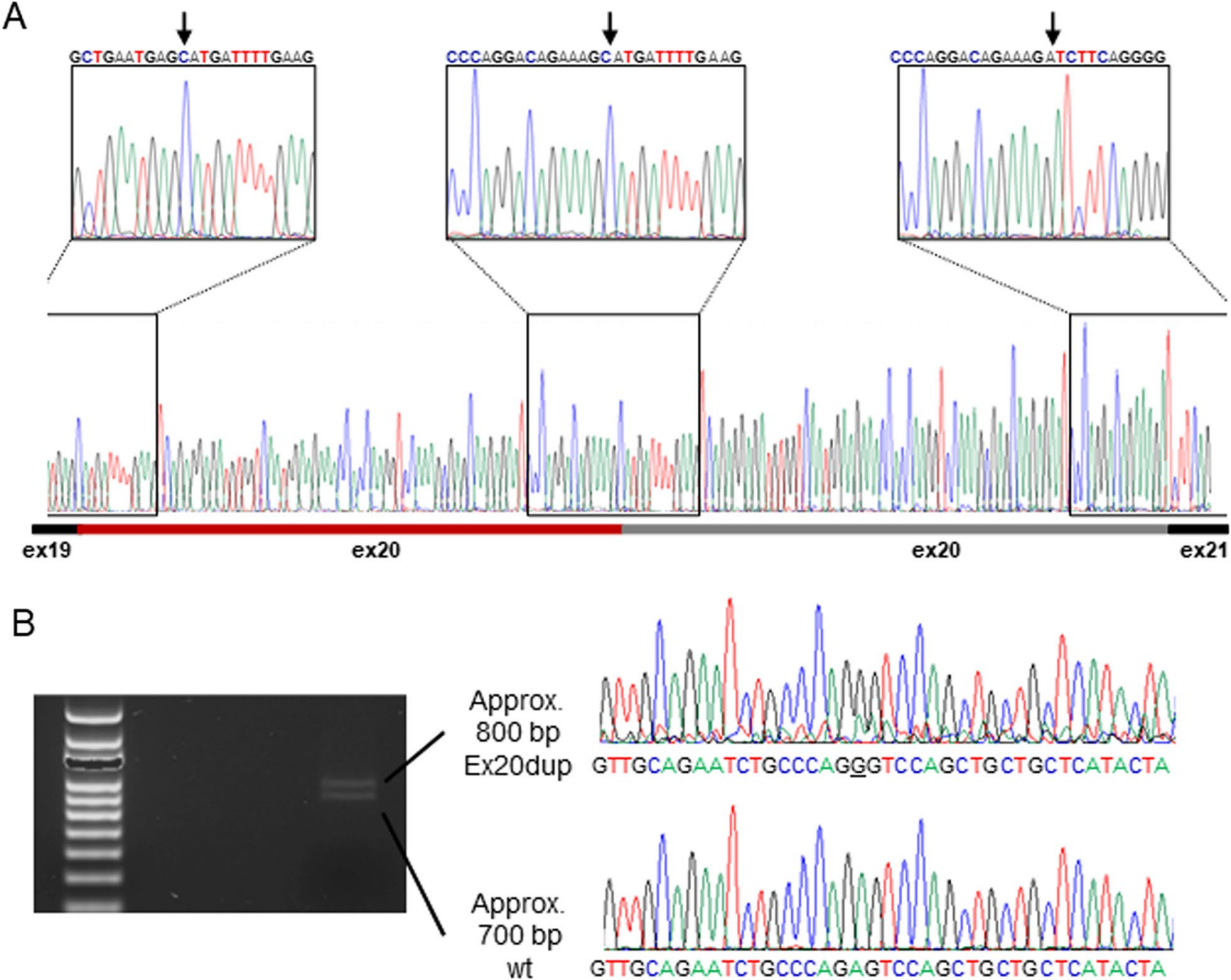
Analysis of the *BRCA1* Ex20dup transcript. **A** Sanger sequencing results of cloned cDNA fragments of Family 1 patient III:1 confirm the expression of an in-frame *BRCA1* Ex20dup transcript. Black arrows in the upper panel indicate exon transitions. **B** PCR-amplification of cDNA retrieved from Family 2 patient IV:2 provides two products spanning from exon 15 to exon 21. A heterozygous single nucleotide polymorphism (rs1799966, underlined) located on exon 16 allows discrimination between the transcripts originating from the wild-type allele (approx. 700 bp) and the *BRCA1* Ex20dup carrying allele (approx. 800 bp). The corresponding uncropped original gel image is shown in Additional file 1: Figure S3

To identify the genomic location of the exon 20 duplication, we performed long-read WGS on DNA of the proband (F1 III:1). Alignment of 50 kb reads supports a tandem duplication with forward orientation (Fig. 2A). To further fine-map the breakpoints, Illumina WGS was performed. As schematically summarized in Fig. 2B, multiple inverted reads mapped to the genomic *BRCA1* location chr17:41,203,000-chr17:41,218,000. Moreover, data analysis using the IGV software suggested a tandem duplication of 5.163 kb region spanning from chr17:41,206,829-chr17:41,211,992 (Additional file 1: Figure S1). Subsequently, we validated the breakpoint by Sanger sequencing (Fig. 2C) indicating location of a breakpoint between chr17:41,206,830-chr17:41,206,840 and chr17:41,211,993-chr17:41,212,003. Since the sequence of 11 bp (GCTCACTGCAA) is identical in these regions, a more precise breakpoint definition was not possible. The formal HGVS nomenclature for the variant is based on this c.5194-2841_5277 + 2229dup. Sanger sequencing of family 2 (F2 IV:2, Fig. 2C) confirmed the same breakpoint; hence, the variants are identical. However, extended pedigree analysis did not link the two families although they stem from the same geographic region in contrast to the family from Italy. In this Italian family, a duplication of an 8706 bp region was reported with breakpoints located in position chr17:41,213,666 and chr17:41,204,961, respectively [12]. Thus, different breakpoints were identified in the Italian family in comparison with the Danish families. To confirm that the duplication of exon 20 (84 bp) results in an in-frame transcript, RNA originating from immortalized lymphocytes of F1 III:1 was reverse transcribed and analyzed. Sanger sequencing of cloned cDNA fragments spanning from exon 16 to 24 confirmed the presence of a *BRCA1* Ex20dup transcript (Fig. 3A, Additional file 1: Figure S2). Moreover, RNA of F2 IV:2 was extracted from blood, reversely transcribed and analyzed by Sanger Sequencing to confirm the presence of a *BRCA1* Ex20dup transcript and to exclude exon 20-related alternative splicing (Additional file 1: Figure S2). The PCR-based amplification of an area spanning from exon 15 to exon 21 generated two products of different lengths (Fig. 3B, uncropped gel image in Additional file 1: Figure S3).

Furthermore, using the same RT-PCR product from F2 IV:2, we also tested the possibility of splicing-rescue i.e. skipping of one of the exon 20 copies by splicing from the Ex20dup allele. We did this by sequencing a heterozygous G/A single nucleotide polymorphism (rs1799966) located in exon 16. This showed that the G nucleotide is present on the allele with the duplication of exon 20 and not detectable on the wild-type allele only displaying the A nucleotide (Fig. 3B).

**Fig. 4:**
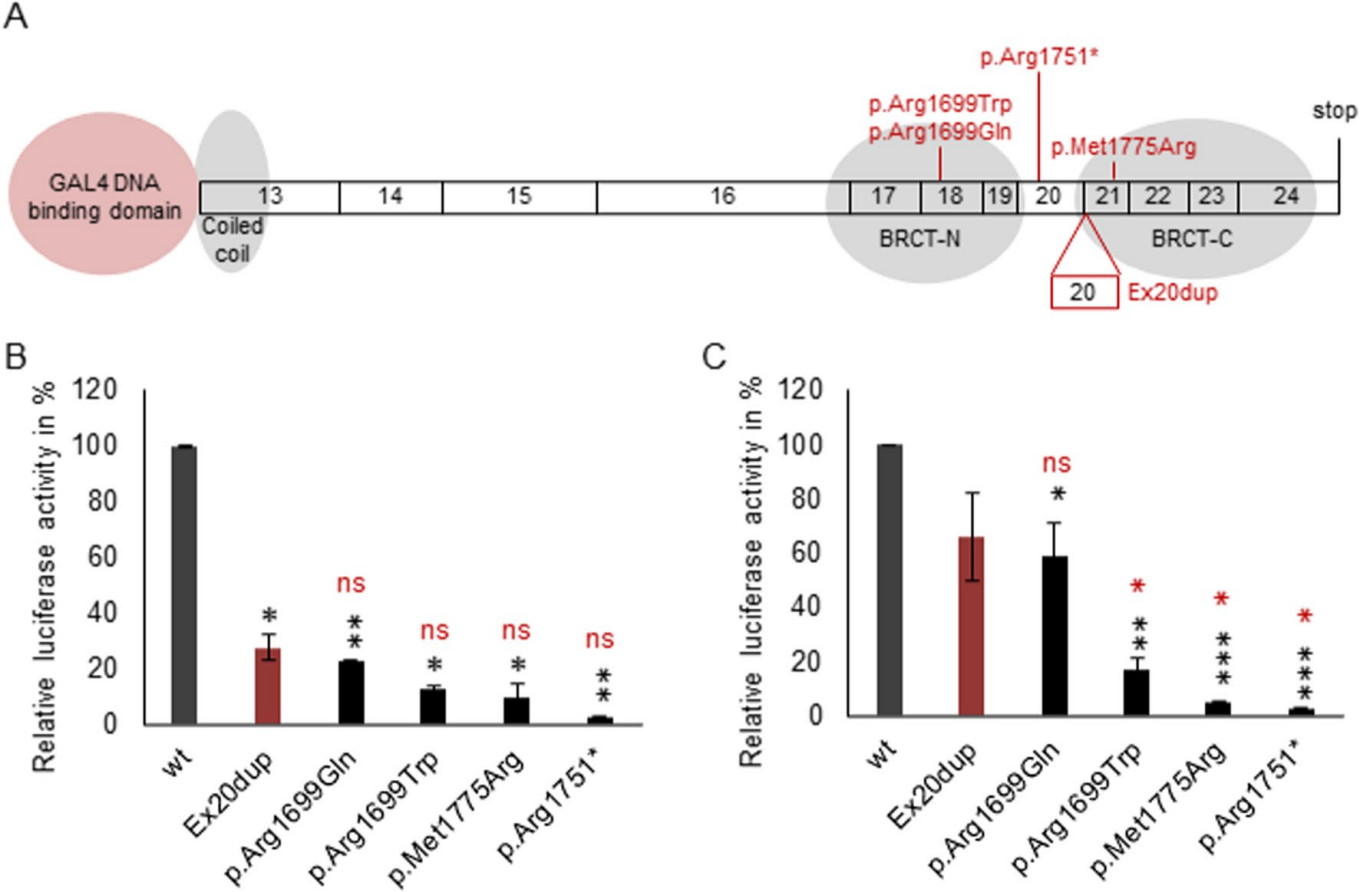
Analysis of the transcriptional activity of the BRCT domains carrying Ex20dup in comparison with wild-type BRCA1 (wt) regions and BRCA1 variants conferring a high (p.(Arg1699Trp), p.(Arg1751*) and p.(Met1775Arg)) or intermediate (p.(Arg1699Gln)) risk for breast and ovarian cancer. **A** Overview of cloned variants (BRCA1 exon 13 to exon 24) fused to the GAL4 DNA binding domain for subsequent Transcriptional Activation assay. The assays were performed in **B** human embryonic kidney cells HEK293 (n = 2) and **C** epithelial breast cancer cells T47D (n = 3). The relative luciferase activity normalized to the BRCT wt domain is shown. Error bars represent standard deviations and significance was determined via an unpaired, two-tailed Student’s t-test. The black stars indicate significance of reduction compared to wt, and the red stars indicate differences between Ex20dup and individual variants with *p < 0.05, **p < 0.01 and, ***p < 0.001, ns: not significant

Finally, we analyzed if the identified *BRCA1* Ex20dup variant would result in a protein with retained C-terminal functionality. Thus, we performed a classical Transcriptional Activation (TA) assay using the human embryonic kidney cell line HEK293 and the epithelial breast cancer cell line T47D [14]. BRCT (BRCA1 Carboxy Terminal) 1–2 domains of BRCA1 were cloned and expressed as a fusion protein with GAL4, and cells were co-transfected with a luciferase reporter vector under a GAL4 promoter to assess transcriptional activation capacity of the BRCT1-2 functional domain. Apart from the wild type and Ex20dup *BRCA1* sequences, four pathogenic variants that also impaired BRCA1 DNA binding ability were analyzed. A schematic overview of the reporter construct and analyzed BRCA1 variants is displayed in Fig. 4A. The known pathogenic control variants p.(Arg1751*), p.(Met1775Arg) [15] and p.(Arg1699Trp) [5] were proven to cause almost complete loss of function, with residual activities ranging from 2.4% to 16.9% in comparison with the wild type *BRCA1* sequence. The p.(Arg1699Gln), known to be associated with a moderate risk relative to an average truncating variant and p.(Arg1699Trp) [16, 17], displayed partial but significant loss of function with 22.6% residual activity in HEK293 cells and 58.7% activity in T47D cells. Comparable values were determined for the Ex20dup variant with 27.3% and 66.2% relative activity in HEK293 and T47D cells, respectively. Thus, the Ex20dup variant exerted a significantly lower transcriptional activity in comparison with the wild-type (Fig. 4B,C), retained a significantly higher activity than the pathogenic control variants p.(Arg1751*), p.(Met1775Arg) and p.(Arg1699Trp), but had similar activity as the moderate penetrance p.(Arg1699Gln) variant. It should be noted that reduction in activity for these two variants was less marked in the hormone sensitive T47D cell line, compared to the HEK293 line.

## Discussion

In this study, we identified a dual *BRCA1* variant male carrier with a tandem duplication of exon 20 and a well-known pathogenic variant c.2475delC *in trans.* At the time of study initiation, the clinical consequence of this in-frame duplication was considered uncertain; it had been reported once in an Italian family where it was suggested to be pathogenic with no further evidence provided for class assignment. However, exon 20 contains part of the BRCT domains, which mediate complex functions of BRCA1 in DNA damage response via, e.g., phospho-protein interactions [18] and in transcriptional activation [19, 20]. Several known pathogenic missense variants have been reported in this domain including c.5213G > A p.(Gly1738Glu) in exon 20, illustrating the importance of this region [21]. We performed allele-specific analysis to eliminate the possibility of rescue of function by exon skipping. Furthermore, we investigated the functional consequences of the duplication by assaying the transcriptional activation using a well-established assay for the BRCA1 BRCT domains [14]. Analysis in both HEK293 and the breast cancer cell line T47D showed that Ex20dup has comparable TAD activity with the missense variant p.(Arg1699Gln), acknowledging increased variability between assay repeats for the T47D assays in particular. The latter variant is well-known to have reduced penetrance compared to average truncating variant, causing risk of breast and ovarian cancer by age 70 years of 20% and 6%, respectively [16, 17, 22].

Evidence from mouse models suggested that one copy of *BRCA1* is necessary for embryonic development [23]. However, recent data have shown that if a minimal level of functional protein is produced from the variant allele, for example due to an alternative isoform rescuing minimal function, the fetus is able to survive. Variants located in exon 11 after nucleotide c.787 are a prominent example because of a naturally occurring in-frame isoform, which lacks the majority of exon 11 (c.788–4096), hence more than half of the gene, and yet retains minimal function in PARP inhibitor and cisplatin resistance assays [6]. All reported dual *BRCA1* carriers present combinations of variants that may retain some protein function. Furthermore, most patients carrying two known presumed “high risk” pathogenic or likely pathogenic variants had some degree of physical features of FA (Table 1).

Our male patient with biallelic *BRCA1* variants had an apparently normal phenotype despite carrying an pathogenic exon 11 frameshift variant and an exon 20 duplication with similar function to a well-known moderate penetrance variant p.(Arg1699Gln). He had few side effects during chemotherapy which also indicates some BRCA1 protein function. Interestingly, the patient reported by Keupp et al*.* carried p.(Arg1699Gln) *in trans* with the p.(Cys61Gly) missense variant, had a mild physical FA phenotype and no detectable chromosomal fragility, but the patient experienced severe chemotoxicity [3]. The p.(Cys61Gly) variant was recently shown to confer a similar risk for early onset of breast and ovarian cancer as protein truncating variants like c.2475delC, but lower risk of breast cancer for patients older than 50 years [5]. Further studies including functional assay analysis may help to identify the minimal level of BRCA1 activity required to avoid FA in dual carriers.

The similarity in transactivating function of the Ex20dup variant to the established reduced penetrance p.(Arg1699Gln) variant could be interpreted to mean that Ex20dup has a similar risk profile to p.(Arg1699Gln), but caution is advisable without insight into the interpretation of experimental and clinical data for reduced penetrance variants in general. The ACMG/AMP guidelines [24], and those specified for *BRCA1/2* (https://cspec.genome.network/cspec/ui/svi/), are designed and calibrated for classifying variants for Mendelian disease. Applying these BRCA1-specified criteria to the Ex20dup variant, there is conflicting evidence toward and against pathogenicity. Criteria applicable include: population frequency data (absence in gnomADSV; PM2_supporting), variant type and location (proven in-frame duplication within domain; PVS1_Strong); lack of recessive Fanconi Anemia phenotype (no physical features, chromosome normal range, no chemotoxicity, cancer age 64y (> 50y); BS2); breast tumor features against pathogenicity (LR 0.32, for ER positive HER2 positive tumor; BP5_Supporting) according to [25]. Regarding segregation analysis, both families were uninformative in this regard: family 1—the variant was from the father’s side without disease and not genotyped, and the carrier son age 49 does not have a disease; family 2—the variant was from the mother’s side and the carrier mother had a cancer type inconsistent with BRCA1-related disease, and no other cancers were reported in the other ungenotyped relatives. Last, although this variant was not previously assayed as part of larger-scale studies accepted for ongoing VCEP use, assay strength of supporting is appropriate based on the Brnich recommendations PS3_Supporting, [26]. Overall, according to a point approach [27], 6 points would be in favor of pathogenicity and 5 points against pathogenicity, and the variant remains of unknown significance. More clinical data, such as that from larger-scale segregation, penetrance and case–control studies, will be required to determine what level of cancer risk may be associated with this specific Ex20dup variant.

We also note the challenges of comparing similar but not identical duplication (or deletion) events. Although an Ex20dup variant was identified in a third family with several cases of breast cancer and lung cancer [13], the breakpoint in the Italian proband was different from the one in our reported Danish families, indicating that a separate analysis of function and clinical studies would be required to investigate its clinical relevance.

## Conclusion

Based on functional data, we propose that the duplication of exon 20 identified in Danish probands may represent a variant with reduced penetrance for breast and ovarian cancer. Further, our results may provide an indication of the level of BRCA1 function that might prevent development of FA physical and chromosomal features in dual carriers. This is an important consideration for classification of *BRCA1* variants of unknown significance, since co-occurrence *in trans* with a known pathogenic variant is often used as an argument for benign classification of variants [28]. The cases reported so far (Table 1) indicate that caution should be taken in this approach depending on the location and type of pathogenic variant.

Our findings may also be informative for risk assessment in potential FA families as it shows that variants with some *BRCA1* function may not predispose to classical FA features.

### Supplementary Information

**Additional file 1. **Supplementary Tables and Figures.

## Data Availability

Data and material analyzed during the study are available from the corresponding author on reasonable request.

## Abbreviations

Ex20dup: Exon 20 duplication
TAD: Transcription activation domain assay
MMC: Mitomycin C assay
FA: Fanconi Anemia
LCL: Immortalizing lymphocytes
MLPA: Multiplexed Ligation-dependent Probe Amplification
WGS: Whole genome sequencing
