# Supplementary material for: Male with an apparently normal phenotype carrying a BRCA1 exon 20 duplication in trans to a BRCA1 frameshift variant
Source: Breast Cancer Res. 2024 Jan 9;26:6. doi: 10.1186/s13058-023-01755-9 (PMC10775606; doi:10.1186/s13058-023-01755-9)
Supplement: Supplementary file 1 — Additional file 1. Supplementary Tables and Figures. [file 13058_2023_1755_MOESM1_ESM.docx]

**Supplementary file**

Male with an apparently normal phenotype carrying a *BRCA1* exon 20 duplication *in trans* to a *BRCA1* frameshift variant

Ines Block^1,2^*, Àngels Mateu-Regué ^3^*, Thi Tuyet Nhu Do^1^*, Ieva Miceikaite^1,4^, Daniel Sdogati^5,6^, Martin J. Larsen^1,4^, Qin Hao^1,4^, Henriette Roed Nielsen^1^, Susanne E. Boonen^1^, Anne-Bine Skytte^7^, Uffe Birk Jensen^7^, Louise K. Høffding^8^, Arcangela De Nicolo^9^, Alessandra Viel^10^, Emma Tudini^11^, Michael T. Parsons^11^, Thomas v. O. Hansen^12,13^, Maria Rossing^3,13^, Torben A. Kruse^1,4^, Amanda B. Spurdle^11^, Mads Thomassen^1,4^

^1^ Department of Clinical Genetics, Odense University Hospital, Denmark

^2^ Current address: Institute of Pharmacology and Clinical Pharmacy, University of Marburg, Marburg, Germany

^3^ Center for Genomic Medicine, Rigshospitalet, Copenhagen University Hospital, Denmark

^4^ Clinical Genome Center, Human Genetics, Department of Clinical Research, University of Southern Denmark, Denmark

^5^ Lundbeckfonden Center of Excellence NanoCAN, Institute of Molecular Medicine, University of Southern Denmark, Odense, Denmark

^6^ Molecular Oncology, Institute of Molecular Medicine, University of Southern Denmark, Odense, Denmark

^7^ Department of Clinical Genetics, Aarhus University Hospital, Denmark

^8^ Center for clinical genetics and genomic diagnostics, Zealand University Hospital, Denmark.

^9^ Center for Omics Sciences, IRCCS San Raffaele Scientific Institute, Milan, Italy

^10^ Unit of Functional Oncogenetics and Genomics, Centro di Riferimento Oncologico di Aviano (CRO) IRCCS, Aviano (PN), Italy

^11^ Population Health Program, QIMR Berghofer Medical Research Institute, Herston, Brisbane, Australia

^12^ Department of Clinical Genetics, Rigshospitalet, Copenhagen University Hospital, Copenhagen, Denmark

^13^ Department of Clinical Medicine, Faculty of Health and Medical Sciences, University of Copenhagen, Copenhagen, Denmark

 *: these authors contributed equally

**
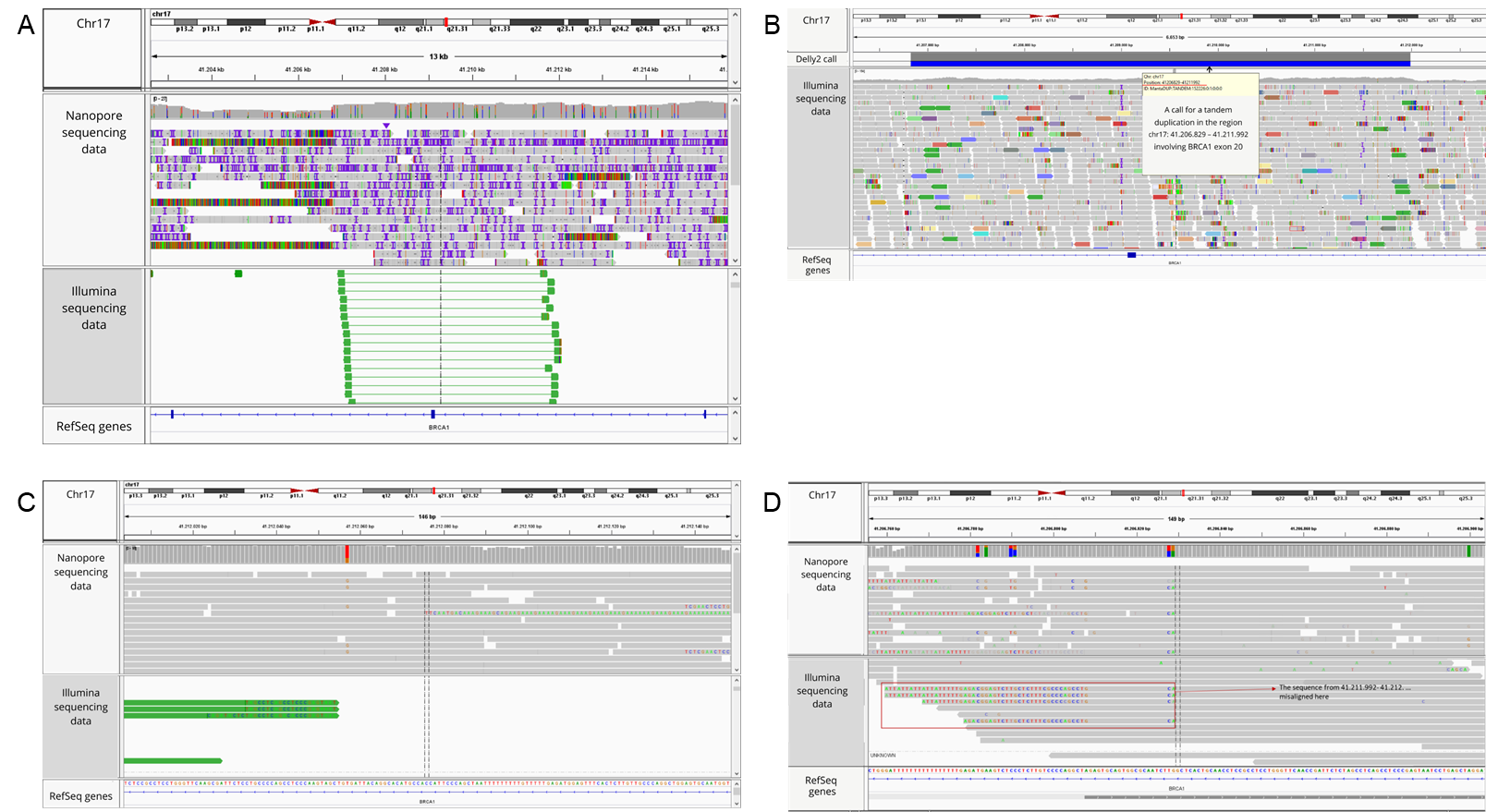
Supplementary Figure S1:** Summary of the data analysis from short-read (Illumina) and long-read (Nanopore) whole genome sequencing visualized in IGV (Integrative Genomics Viewer) for proband F1 III:1. (A) The screenshot showing sequencing data for the structural variant. The mismatched bases (colorful bases in Nanopore seq) and discordant read pairs (green, in Illumina seq) indicate the alignment of the duplicated regions in the same location. The inverted read pairs (Illumina, in green) indicate the tandem duplication. (B) The variant caller software Delly2 identified a tandem duplication which spans chr17:41.206.829-chr17:41.211.992. (C) Visualization of split reads with mismatched bases (colorful bases) supporting the potential breakpoint. (D) Both Illumina and Nanopore data support the breakpoint. However, fine-mapping of the breakpoint was impossible due to the identical sequences of the flanking regions of the breakpoint.


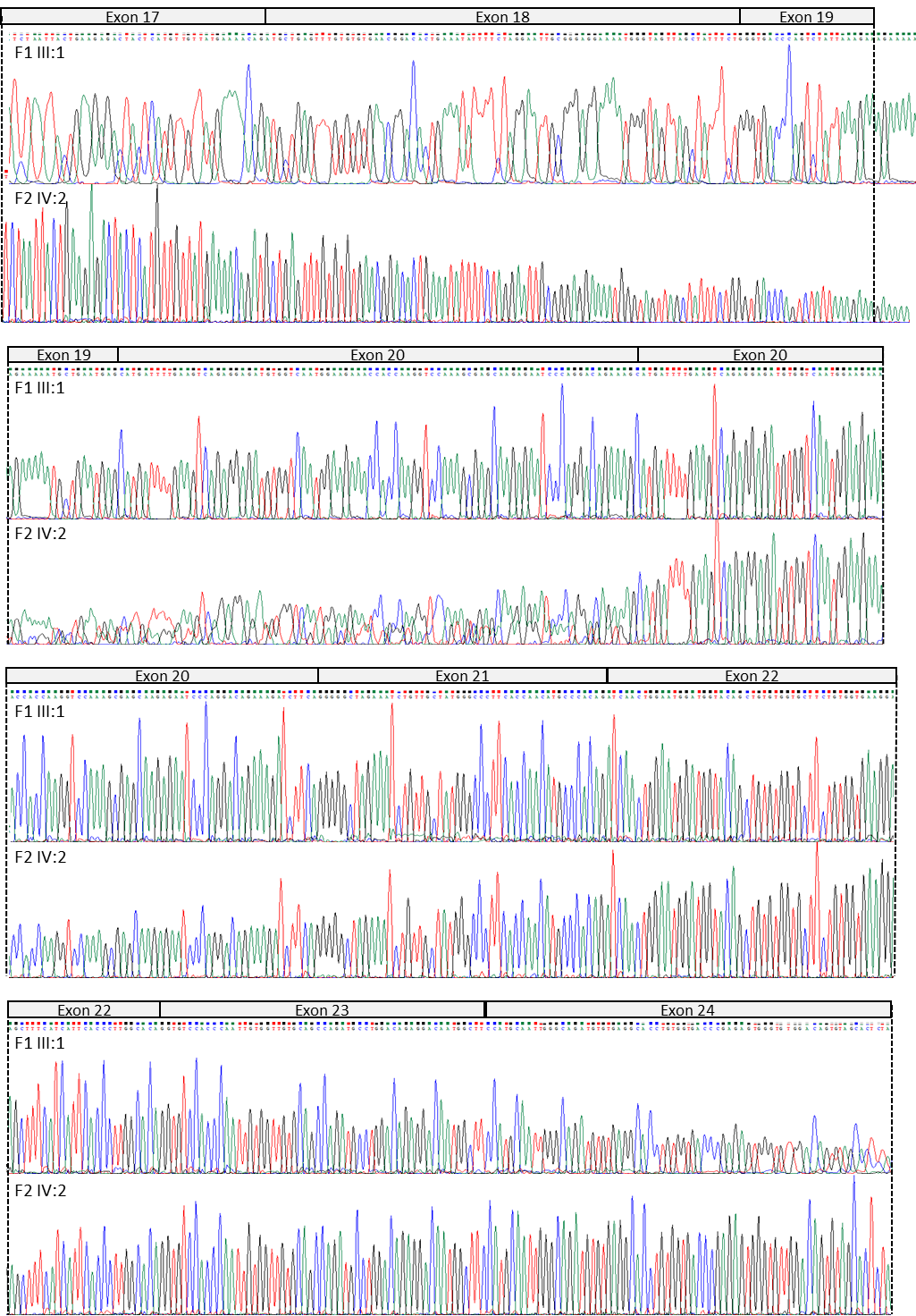


**Supplementary Figure S2:** Sanger sequencing of PCR-amplified and/or cloned Ex20 dup transcripts of proband F1 III:1 and F2 IV:2 spanning exon 17 to exon 24. Overlapping sequences or transition of reads are indicated by the dashed line.


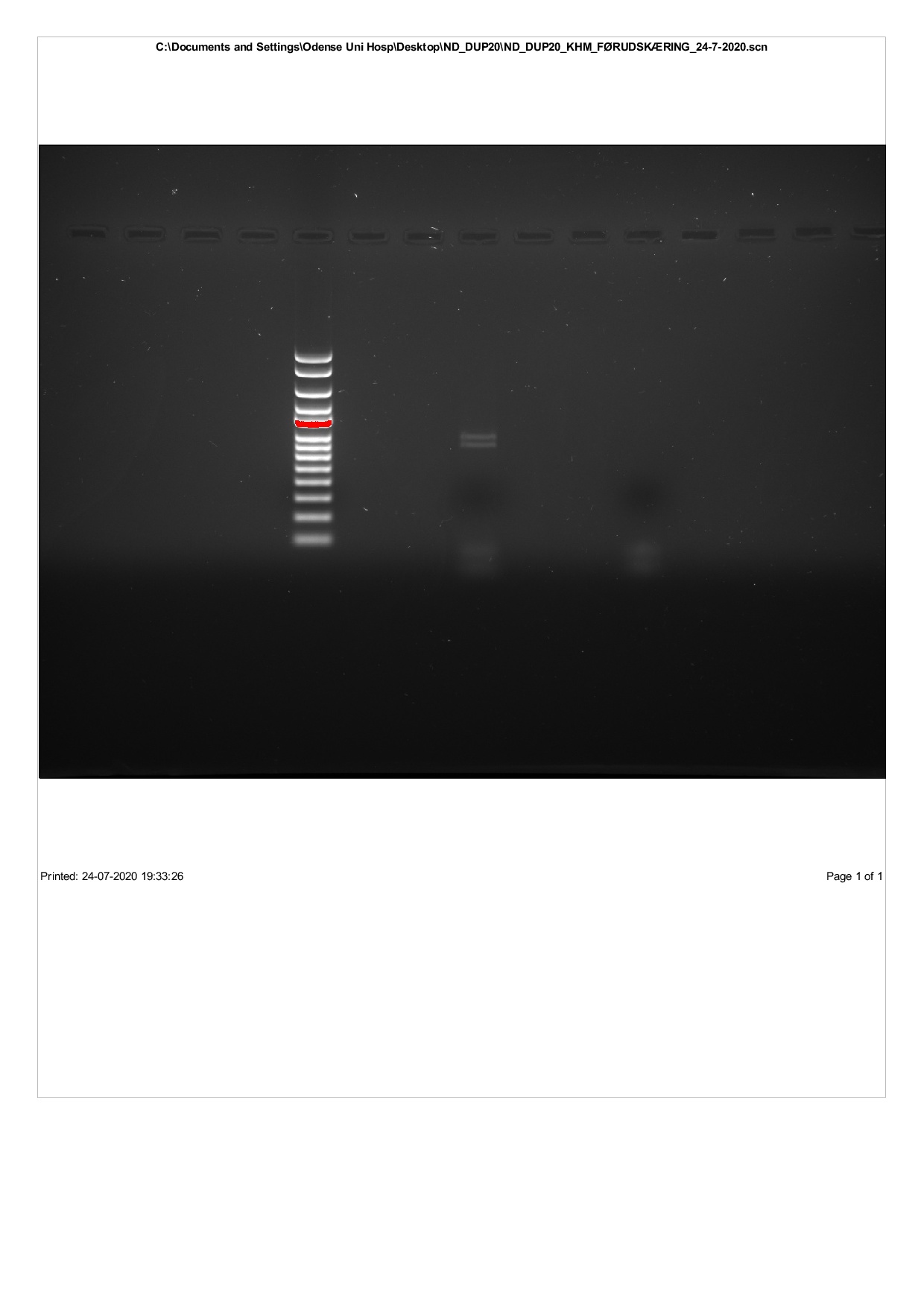


**Supplementary Figure S3:** A section of the uncropped gel image shown here is shown in Figure 3B.

| **Supplementary Table S1:** Sequencing primer used in the study | | | |
| --- | --- | --- | --- |
| **Name** | **Sequence** | **Location** | **Application** |
| BRCA1_Ex16F | 5'-GGGAGAAGCCAGAATTGACA-3' | Exon 16 | Transcript sequencing F2 IV:2, Supplementary Figure S2 |
| BRCA1_Ex24R | 5'-CCTGGAAAGGCCACTTTGTA-3' | Exon 24 | Transcript sequencing F2 IV:2, Supplementary Figure S2 |
| BCe13forw | 5'-ATACCATGCAACATAACCTGATAAAG-3' | Exon 13 | Cloning of fragment F1 III:1 |
| BCe24rev | 5'-GCTGTGGGGGATCTGGGGTAT-3' | Exon 24 | Cloning of fragment F1 III:1; Figure 3A, Supplementary Figure S2 |
| BCe1920forw | 5'-GAATGAGCATGATTTTGAAGTC-3' | Exon 19-20 junction | Cloning of fragment F1 |
| BCe16forw | 5'-AGGGAACCCCTTACCTGGAATCT-3' | Exon 16 | Cloning of fragment F1 |
| BCe16rev | 5'-TCTGATGTGCTTTGTTCTGGATTTC-3' | Exon 16 | Cloning of fragment F1 |
